# MRI analysis to map interstitial flow in the brain tumor microenvironment

**DOI:** 10.1063/1.5023503

**Published:** 2018-06-26

**Authors:** Kathryn M. Kingsmore, Andrea Vaccari, Daniel Abler, Sophia X. Cui, Frederick H. Epstein, Russell C. Rockne, Scott T. Acton, Jennifer M. Munson

**Affiliations:** 1 Department of Biomedical Engineering, University of Virginia School of Medicine, Charlottesville, Virginia 22904, USA; 2 Department of Electrical and Computer Engineering, University of Virginia School of Engineering and Applied Science, Charlottesville, Virginia 22904, USA; 3 Division of Mathematical Oncology, City of Hope, Duarte, California 91010, USA; 4 Department of Biomedical Engineering and Mechanics, Virginia Polytechnic Institute and State University, Blacksburg, Virginia 24061, USA

## Abstract

Glioblastoma (GBM), a highly aggressive form of brain tumor, is a disease marked by extensive invasion into the surrounding brain. Interstitial fluid flow (IFF), or the movement of fluid within the spaces between cells, has been linked to increased invasion of GBM cells. Better characterization of IFF could elucidate underlying mechanisms driving this invasion *in vivo*. Here, we develop a technique to non-invasively measure interstitial flow velocities in the glioma microenvironment of mice using dynamic contrast-enhanced magnetic resonance imaging (MRI), a common clinical technique. Using our *in vitro* model as a phantom “tumor” system and *in silico* models of velocity vector fields, we show we can measure average velocities and accurately reconstruct velocity directions. With our combined MR and analysis method, we show that velocity magnitudes are similar across four human GBM cell line xenograft models and the direction of fluid flow is heterogeneous within and around the tumors, and not always in the outward direction. These values were not linked to the tumor size. Finally, we compare our flow velocity magnitudes and the direction of flow to a classical marker of vessel leakage and bulk fluid drainage, Evans blue. With these data, we validate its use as a marker of high and low IFF rates and IFF in the outward direction from the tumor border in implanted glioma models. These methods show, for the first time, the nature of interstitial fluid flow in models of glioma using a technique that is translatable to clinical and preclinical models currently using contrast-enhanced MRI.

## INTRODUCTION

The tumor microenvironment (TME) consists of all cells, extracellular matrix, chemical factors, and biophysical forces aside from the tumor cells. Together, these components create the complete cancer tissue that is both affected by the cancer and can in turn affect the tumor cells.^1,2^ The TME has been implicated in therapeutic response, invasion, proliferation, and differentiation of tumor cells. In glioblastoma (GBM), a highly aggressive brain cancer, the microenvironment is known to contribute to the invasion of tumor cells into the surrounding healthy brain.^3^ This invasion is partially responsible for poor survival seen in patients, as invaded tumor cells cannot be reached by the current standard of care therapy targeting the tumor bulk. Thus, the identification and characterization of mediators of tumor cell invasion could aid in the treatment of GBM.

We and others have identified interstitial fluid flow (IFF) as an integral component of the tumor microenvironment.^4–10^ *In vitro* analyses using microfluidic devices and tissue culture chambers have shown that IFF is involved in increasing proliferation, triggering invasion of tumor cells, and altering the surrounding microenvironment to promote cancer progression. Growing tumors are marked by increased interstitial pressure, due to accumulation of proliferating tumor cells, extracellular matrix, and fluid, which is higher than the pressure in the surrounding tissue.^11^ This pressure differentially yields increased IFF across the invasive edges of tumors where tumor meets healthy tissue. While it is a potent driver of invasion in brain,^4,5,12^ skin,^13^ hepatic,^6^ and breast cancer,^7,9,14,15^ IFF has been poorly measured and characterized *in vivo*. It is thereby pertinent to develop accurate means to quantify IFF in the pre-clinical and clinical settings.

Chary and Jain pioneered the use of intravital fluorescence recovery after photobleaching (FRAP) to approximate fluid velocity in the rabbit ear.^16^ However, as FRAP requires optical access, measurements are confined to superficial locations and cannot be acquired quickly across the entire tumor and surrounding microenvironment. Butler *et al.* implanted micropore diffusion chambers downstream of breast tumors to measure total fluid drainage.^17^ While micropore chambers provide good measurements of bulk fluid movement, this method does not afford information on interstitial flow velocities and is difficult to implement in most models. Noninvasive attempts to characterize bulk fluid transport *in vivo* employ magnetic resonance imaging (MRI). These approaches in implanted brain (intradermal/subcutaneous) and breast tumors (orthotopic) have used multi-compartment models to approximate IFF velocities based on the rate of change of the contrast-enhanced ring at the tumor border over time,^18^ or identify the fluid drainage volume and pooling rates.^19^ Similarly, other dynamic MRI approaches have estimated fluid velocities in implanted tumor models using equations relating signal intensity to a linear attenuation coefficient.^13,20^ Our goal was to improve and expand these techniques by developing a novel methodology to noninvasively measure IFF directly *in vivo* in GBM. Dynamic contrast-enhanced MRI (DCE-MRI) has been used clinically as a standard imaging method to assess the vascularization of tumors by analyzing the influx of T1 contrast agents (i.e., Gadolinium chelates) and tumor permeability. In GBM alone, DCE-MRI has been used for grading tumors,^21^ discriminating between tumor and radiation necrosis regions,^22^ and predicting survival time of patients.^23^ Here, we take advantage of this common contrast-enhanced MRI technique, to develop our computational methodology to measure IFF in and around brain tumors. Unlike other approaches, we aim to evaluate flow velocities on the basis of biological transport principles. We test our method on several *in vitro* and *in silico* phantoms and apply it to four human stem cell xenograft models of GBM. We quantify and map interstitial flow in these models, relating the patterns of flow to a common marker of fluid drainage from tumors, Evans blue dye, to verify that this method is a valid approach for determining regions of interstitial fluid flow in the brain tumor microenvironment.

## RESULTS

### Spin echo-MRI reveals heterogeneous contrast dynamics

**FIG. 1.**
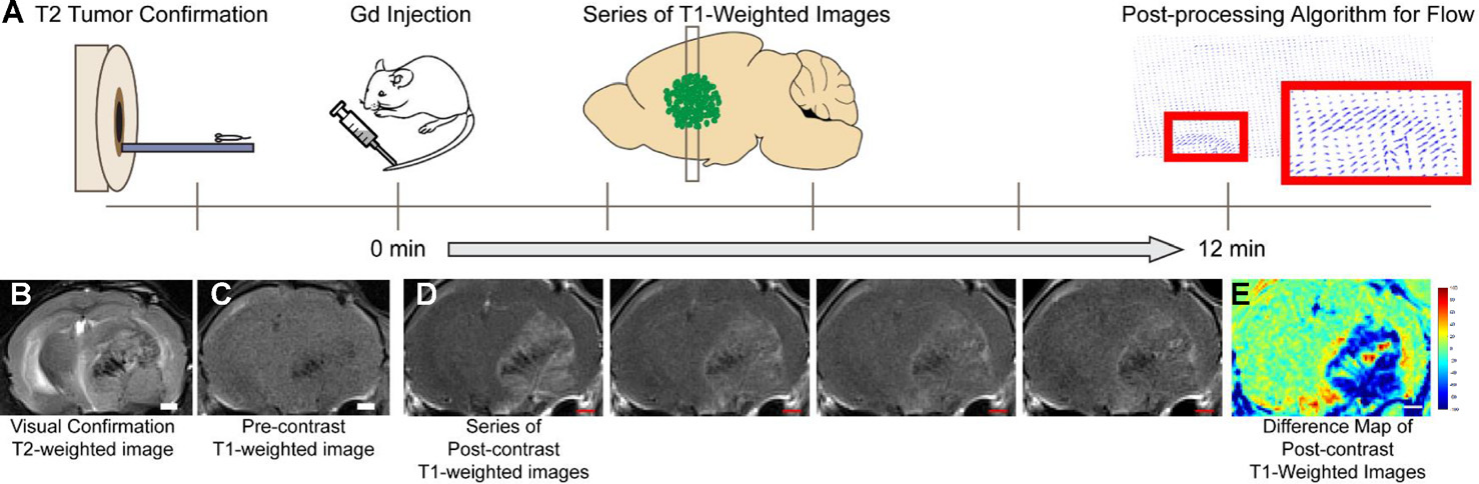
MRI sequence to detect interstitial flow. Spin echo sequence reveals differences in gadolinium intensity over time. (a) Overview schematic of MRI acquisition procedure for fluid flow measurements including (b) tumor confirmation with T2-weighted image followed by (c) pre-contrast T1-weighted image, (d) post-contrast series of T1-weighted images, and (e) difference map between post-contrast image 4 and post-contrast image 1. Scale bar = 1 mm.

We utilized MRI to observe contrast dynamics within our tumor models [Fig. 1(a)]. After T2-weighted confirmation and localization of xenograft tumor [Fig. 1(b)] and pre-contrast T1-weighted image for background subtraction [Fig. 1(c)], a bolus injection of gadobenate dimeglumine (Gd) (Multihance, Bracco Diagnostics) was given. Initial influx of intravenous Gd into the tumor was detected by dynamic contrast-enhanced MRI (supplementary material, Video 1), followed by spin echo MRI (T1-weighted) [Fig. 1(d), supplementary material, Video 2]. Four T1-weighted images were successively taken following injection of Gd to identify tissue transport of extravasated contrast agent [Fig. 1(d)]. This technique was found to provide high resolution imaging over our desired time course. Difference maps of final-initial contrast agent localization were used to confirm transport of Gd over the duration of imaging [Fig. 1(e)].

### Mathematical model of Gd transport allows us to probe flow field

Based on our dynamic magnetic resonance (MR) sequences, we wished to determine the velocities of interstitial flow *in vivo.* Biological transport principles govern interstitial flow, and we proposed to approximate fluid velocity by assuming that it was equal to the velocity computed for solute movement. We described the movement of our solute, Gd, within the tumor and adjacent brain tissue as a convective process that combines microscopic diffusion and macroscopic bulk motion. As described in more detail in the Methods section, we built a mathematical model of the spatiotemporal evolution of solute, Gd [Eq. (1)], which allowed us to computationally solve the inverse problem, i.e., to estimate the flow velocity field that best explains the observed change in MR signal intensity caused by transport of the Gd contrast agent.

### *In vitro* and *in silico* models determine the sensitivity of our computational approach

To evaluate our parameter estimation approach, we took advantage of our previously described *in vitro* hydrogel-tissue culture insert system, in which we could directly measure average velocity,^24^ and numerical simulations in which we could control the velocity magnitude and direction in a control volume.

#### In vitro *model*

**FIG. 2.**
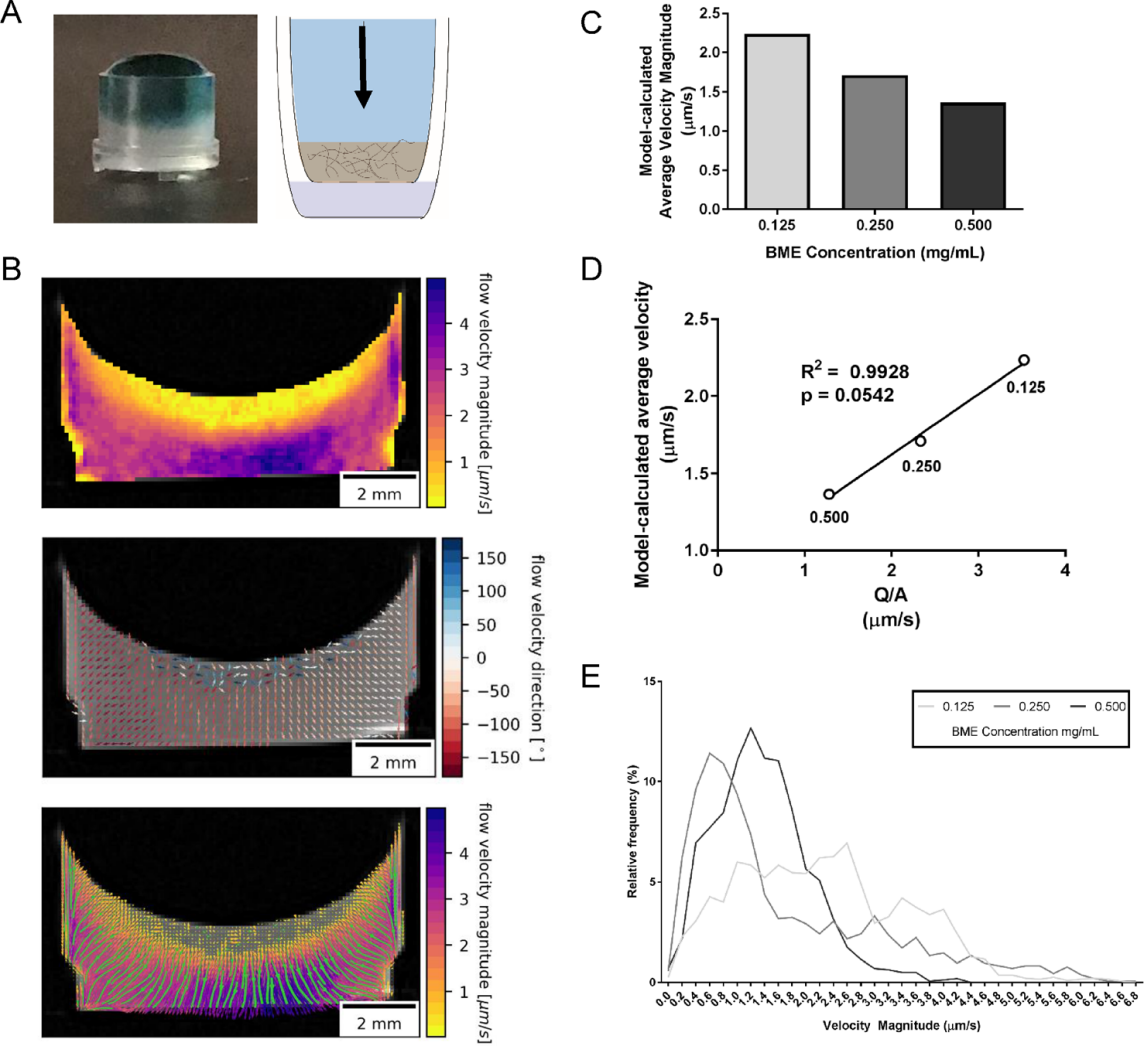
*In vitro* phantoms validate our computed direction and average velocity. (a) Picture (left) and schematic (right) of our tissue culture insert system: a collagen I-basement membrane extract (BME) hydrogel is seeded atop the porous insert and pressure driven flow moves media containing gadolinium (blue) through the gel (beige). (b) Reconstructed flow velocity magnitude (top), velocity direction (middle), and velocity magnitude with quiver plots (bottom) through the *in vitro* tissue culture insert system with velocity magnitude ranging from 0 to 4.5 *μ*m/s with downward velocity vectors. (c) Model-calculated average velocity through *in vitro* tissue culture insert system relative to BME concentration. (d) Correlation between model-calculated average interstitial flow velocity magnitude and manually measured average flow velocity (Q/A). (e) Histogram of model-calculated interstitial velocity magnitudes with different BME gel constructions.

We have optimized our tissue culture insert system to study pressure-driven flow through a tissue-mimicking hydrogel.^24,25^ This system consists of a tissue culture insert with a collagen I gel and varying concentrations of basement membrane extract (BME) to tune the overall volumetric flow [Fig. 2(a)]. By applying a pressure head atop the hydrogel, fluid is driven through the gel and into the lower chamber over the course of 18 h. We imaged this system as the contrast agent moved through the gel over time during the washout of the agent with non-contrast containing media, after addition of Gd to the upper chamber. MR and analysis revealed that Gd flow exhibited velocities between 0 and 4.5 *μ*m/s [Fig. 2(b), top panel] and was primarily downward directed [Fig. 2(b), middle and bottom panel] which was the expected direction. Unexpected variability in estimated flow directions in the upper central part of the gel is likely due to early washout of Gd in that region [Fig. 2(b), middle panel]. As a result of washout, little to no change in MR signal intensity is detectable during subsequent image acquisitions, limiting the ability of our reconstruction algorithm to reliably identify optimal parameters. By selecting a time window within the MR images where the concentration of Gd was changing throughout the course of acquisition, we could determine an average velocity magnitude through the hydrogel. Using gels with increased concentration of BME reduced the model-calculated average velocity magnitude [Fig. 2(c)], in line with previously published studies.^7^ Similarly, average model-estimated diffusivity decreased as BME concentration increased, with diffusivity ranges comparable to those previously reported for collagen gel systems [supplementary material Figs. 1(a) and 1(b)].^26^ Average model-estimated velocity magnitudes ranged between 1.37 and 2.24 *μ*m/s [Fig. 2(c)] for the different gels and were significantly correlated to manually measured average velocities (Q/A) of 1.17–3.52 *μ*m/s in the same systems [Figs. 2(d) and 2(e)].

#### In silico *model*

**FIG. 3.**
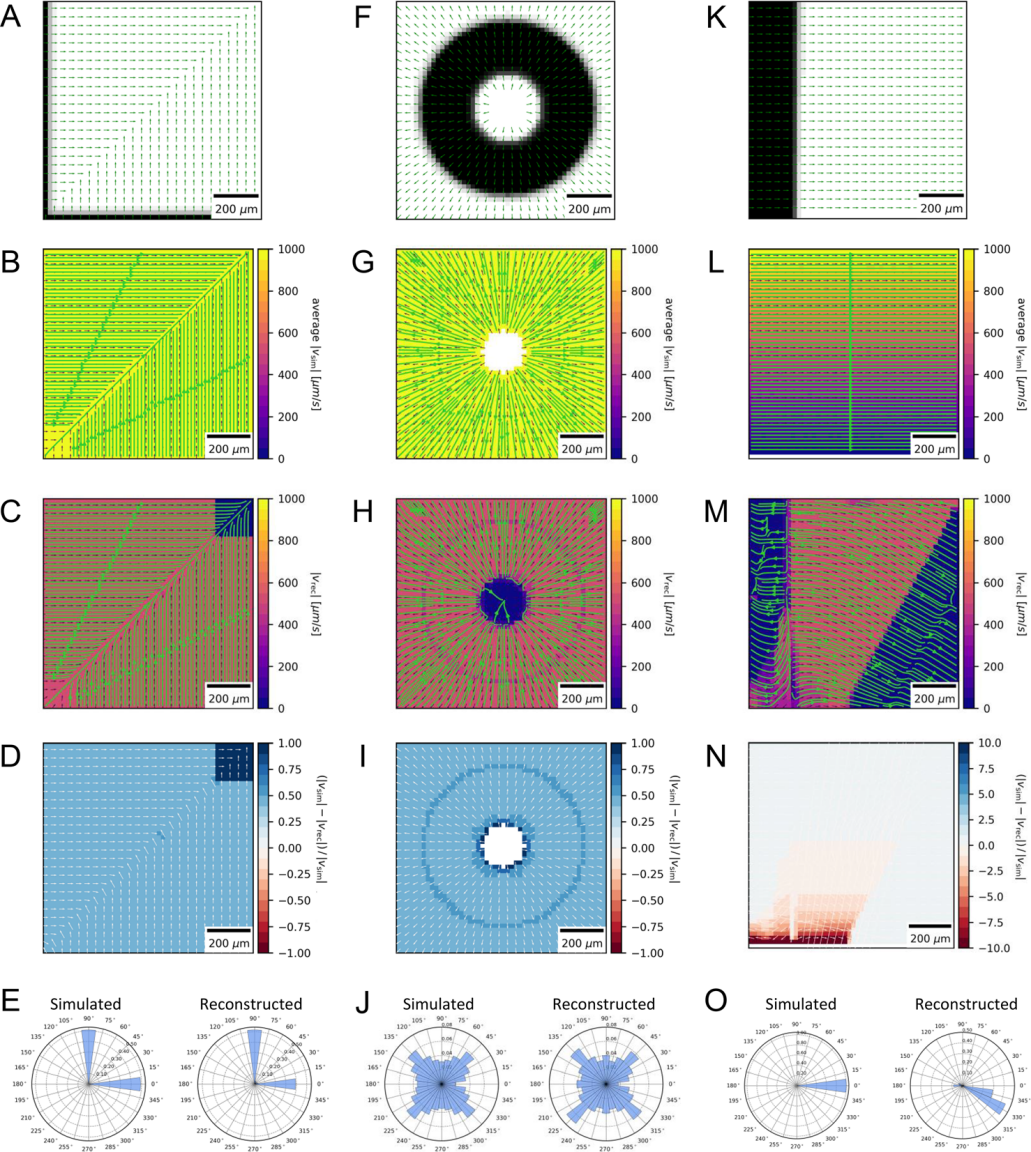
*In silico* phantoms evaluate the sensitivity of our model. (a) Schematic of, (b) simulated velocity field for, and (c) reconstructed velocity magnitude map with streamlines of bidirectional flow velocity of constant magnitude. Overall comparison of (d) velocity magnitude and (e) direction between simulation (left) and reconstruction (right). (f) Schematic of, (g) simulated velocity field for, and (h) reconstructed velocity magnitude map with streamlines of multidirectional flow of constant magnitude. Overall comparison of (i) velocity magnitude and (j) direction between simulation (left) and reconstruction (right). (k) Schematic of, (l) simulated velocity field for, and (m) reconstructed velocity magnitude map with streamlines of unidirectional flow with spatially varying magnitude. Overall comparison of (n) velocity magnitude and (o) direction between simulation (left) and reconstruction (right).

To further test our estimation approach, we computationally solved the forward model [Eq. (1)] for specific flow scenarios, which allowed us to compare the estimated to actual flow parameters. We generated computational phantoms mimicking idealized flow situations of bidirectional [Figs. 3(a) and 3(b)] and multidirectional (360°) flow [Figs. 3(f) and 3(g)] of spatially constant magnitude, and unidirectional flow of spatially varying magnitude [Figs. 3(k) and 3(l)]. Evaluation of our reconstruction approach on these *in silico* phantoms showed that the algorithm performs well on flow fields of spatially constant velocity magnitude [Figs. 3(b) and 3(g)]. The direction of flow was reconstructed correctly, locally throughout the domain [Figs. 3(c) and 3(h)] as well as globally [Figs. 3(e) and 3(j)]. Flow velocity magnitude was consistently reconstructed to approximately 50% of the simulated velocity, except for boundary regions where a larger relative error was observed. The algorithm performed less well on fields with spatially variable velocity magnitude [Fig. 3(l)], where it overestimated the flow velocity magnitude in regions of slower flow [Figs. 3(m) and 3(n)] up to a factor of greater than or equal to 10 at the domain boundary. However, despite differences in the velocity magnitude and local velocity direction [Fig. 3(m)], the overall flow direction in this scenario was reconstructed within ±30° [Fig. 3(o)].

### Interstitial fluid flow velocities in GBM are heterogeneous within a single tumor

**FIG. 4.**
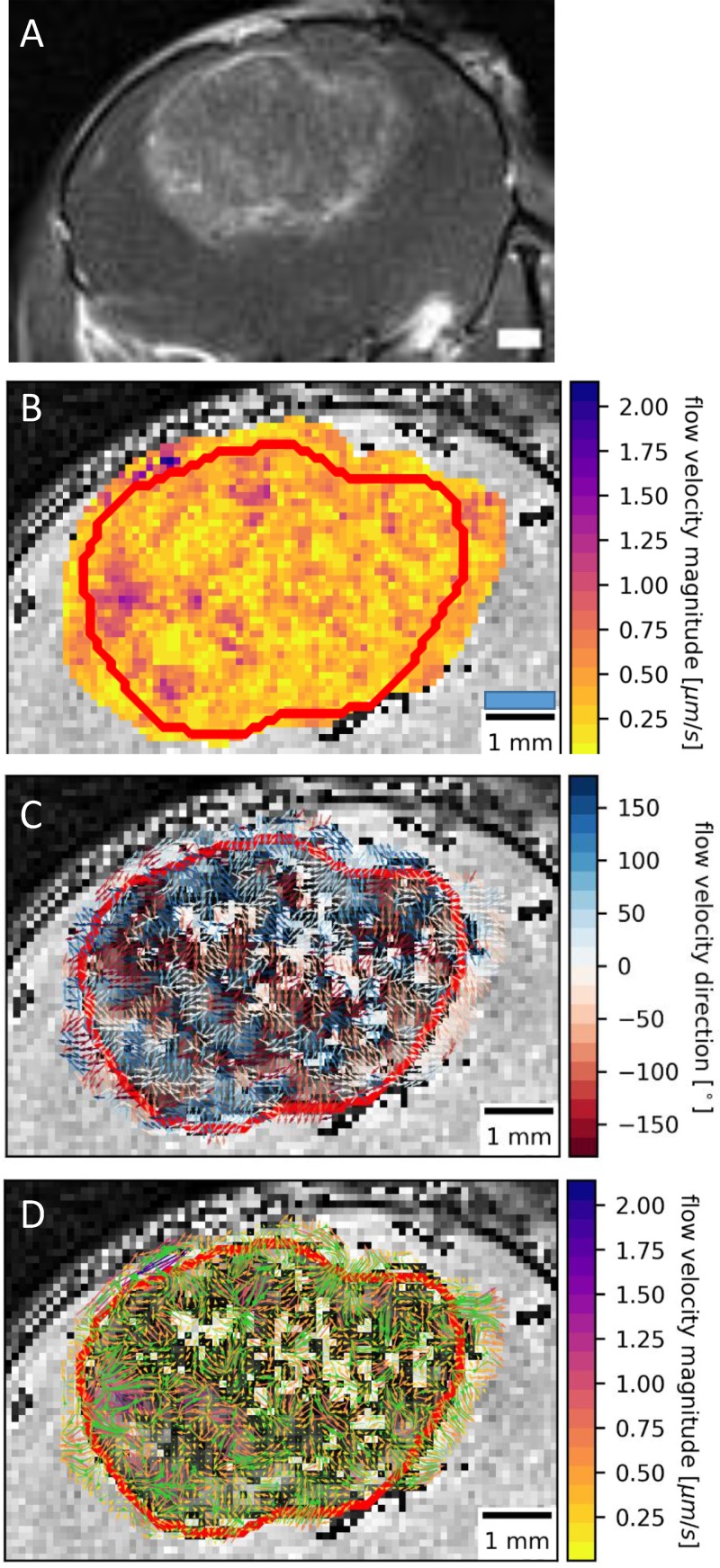
*In vivo* heterogeneity of interstitial fluid flow in glioblastoma. (a) T1-weighted MRI with contrast used for identification of tumor analysis region, scale bar = 1 mm. (b) Velocity magnitude output, (c) velocity vector output, and (d) velocity magnitude map overlaid with velocity vector plots from computational analysis in the selected region with the tumor border shown in red.

**FIG. 5.**
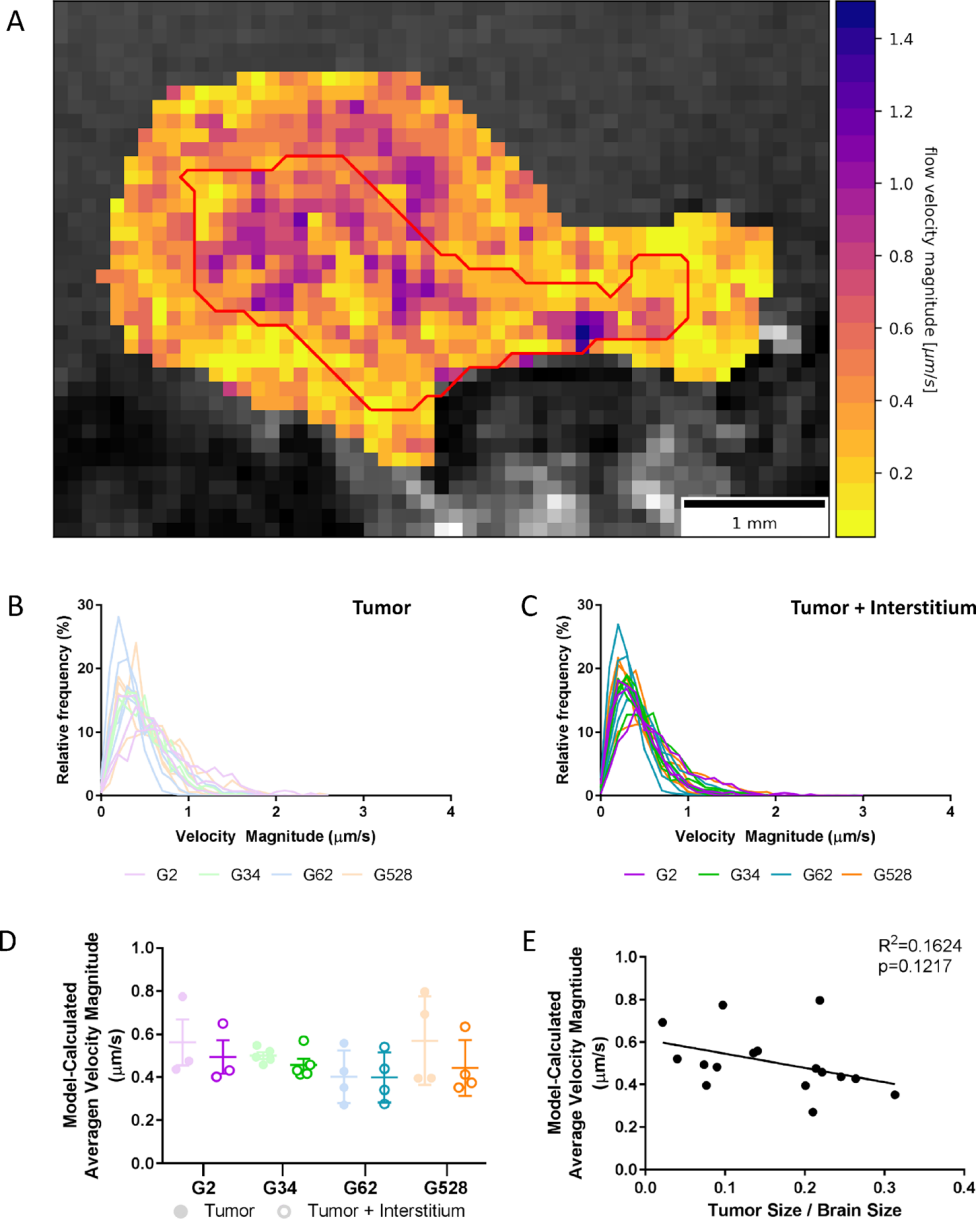
Histograms of interstitial velocities reveal similar ranges across tumor models. (a) Velocity magnitude output map showing the selected region of tumor (red outline) or tumor + interstitium, the entire region. (b) Histogram of velocity magnitude within the tumor for G2, G34, G62, and G528. (c) Histogram of velocity magnitude within and around the tumor for G2, G34, G62, and G528. (d) Average model-calculated interstitial velocity magnitude within and around the tumor. (e) Model-calculated average tumor velocity magnitude correlated with the normalized tumor size.

Using our MRI-based flow reconstruction approach, we aimed to characterize and quantify interstitial velocities in and around murine xenografts of glioblastoma stem cell (GSC) lines. Either the tumor alone, or combined tumor and surrounding interstitium, where we saw changes in the contrast agent, was first demarcated on the first post-contrast T1-weighted image to identify the region for analysis [Fig. 4(a)]. We then applied our reconstruction approach in this region to estimate IFF velocity maps from which velocity magnitude [Fig. 4(b)] and direction [Fig. 4(c)] were derived. Both the velocity magnitude and direction were highly variable across each individual tumor, indicating a heterogeneous frequency of interstitial velocity that was not always in the outward direction [Fig. 4(d)]. The distribution of velocity magnitudes within each tumor was positively skewed and, interestingly, was similar between tumor models [Figs. 5(a) and 5(b), supplementary material Figs. 2(a)–2(d)]. This was also true when the region outside of the tumor bulk was included (the interstitial region) [Figs. 5(a) and 5(c), supplementary material Figs. 2(a)–2(d)]. The distribution of diffusivities was normally distributed, but again similar between models [supplementary material Figs. 3(a), 3(b), and 4]. Comparison of the tumor bulk interstitial velocity magnitudes to the tumor bulk + interstitial space indicated that the average flow rates in these interstitial regions were not significantly different from those in the bulk alone [Fig. 5(d)]. Neither the mean [Fig. 5(d)] nor the median [supplementary material Fig. 2(e)] calculated interstitial velocity magnitudes were significantly different between tumor models. Similarly, neither the mean nor the median calculated diffusivities were significantly different between tumor models, except the average bulk diffusivity between G62 and G528 in which G528 was significantly higher [supplementary material Figs. 3(c) and 3(d)]. Since there was a high degree of variability between the tumor-bearing mice, we decided to examine the correlation of outcomes on a mouse-by-mouse basis to identify possible contributing factors to interstitial flow velocity differences. The tumor size was variable at the same timepoint across models, but, we did not observe a significant correlation between the tumor size and the mean interstitial velocity magnitude across tumor models [Fig. 5(e)], nor was there a correlation between the average model-calculated velocity magnitude and average model-calculated [supplementary material Fig. 2(f)]. Thus, it appeared that intratumoral heterogeneity was more apparent in the four tumor models we examined, than intertumoral variability.

### Traditional markers of fluid drainage correlate with our flow patterns

**FIG. 6.**
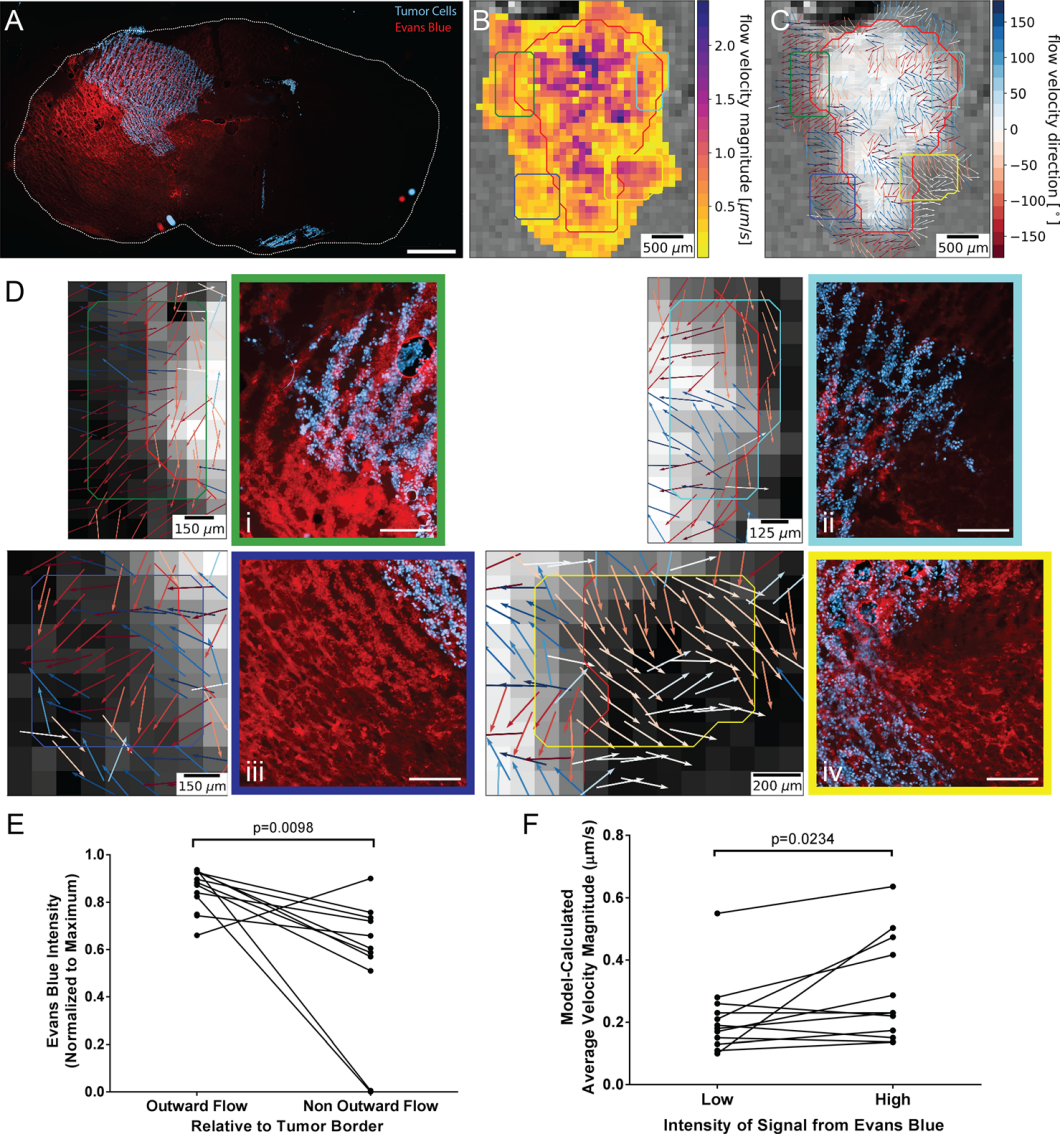
Directional mapping of interstitial flow correlates with Evans blue intensity. (a) Whole-histological section scan of brain (white dotted line) with tumor (human nuclear antigen, cyan) and Evans blue (red), scale bar = 1 mm. Boxes denote the corresponding locations on MRI. (b) Velocity magnitude map in the user-selected region of tumor (red outline) or tumor + interstitium, the entire region generated from computational analysis. (c) Velocity vector map within the same region with arrows indicating direction of flow. (d) High magnification images of velocity vectors (left) and histological sections [right; tumor cells (cyan) and Evans Blue (red)] for corresponding regions from A to C; scale bar = 100 *μ*m (e) Evans blue intensity as measured in histological sections in regions with generalized outward flow vs non-outward flow. Each point in graphs is an average for a single mouse across multiple sampled areas per tumor. Analyzed by paired t-test with non-parametric distribution correction. (f) Average velocity magnitude in regions of low Evans blue intensity vs high intensity within single mice, averaged across multiple areas per tumor. Analyzed by paired t-test with non-parametric distribution correction.

Evans blue and other tracer dyes have been used for decades to determine the movement of fluid from tumors.^27^ We have used it frequently to identify histological regions of interstitial fluid flow within the tumor microenvironment.^4,5^ Upon intravenous injection, Evans blue binds to albumin in the bloodstream and only crosses into the brain through compromised tumor-associated vasculature, thus allowing us to uniquely examine transport of this molecule from the tumor into surrounding parenchyma, similar to Gd. To determine how our reconstruction approach matches with this histological method, we wished to correlate Evans blue intensity with our calculated outcomes. We hypothesized that regions with higher Evans blue intensity would have both a net outward direction to the velocity vector fields and higher velocity magnitudes than in regions of lower Evans blue intensity. Multiple user-defined regions of Evans blue positivity were selected [Figs. 6(a)–6(c)] and defined as high positivity if the intensity of the region was greater than 2/3 of the maximum integrated density measured for the tumor, similar to previous approaches we have used.^5^ We also normalized intensity to the maximum intensity measured for the tumor. Then, the average interstitial velocity of the region was computed using our method on the matched MRI section [Figs. 6(b) and 6(c)]. Flow patterns were grouped into either outward flow (±45° from the normal to the tumor border at that point) or non-outward (indicating either inward flow, or parallel flow) with regard to the tumor border. Regions with outward flow [Fig. 6(d)-i, iii, and iv] were characterized by higher Evans blue intensity in histological sections when compared with the integrated density in regions of non-outward flow [Fig. 6(d)-ii] for 10 out of 12 tumors analyzed [Fig. 6(e)]. Calculation of the average velocity magnitude in these regions indicated that regions with high Evans integrated density showed average velocity magnitudes that were higher than those in low Evans blue integrated density [Fig. 6(f)]. Thus, our flow patterns validate the histological outcome of Evans blue drainage within and around the tumor.

## DISCUSSION

### Measurement of interstitial flow

The biophysical parameter of IFF velocity has not been widely studied *in vivo*, though *in vitro* analyses suggest that it is an important contributor to tumor cell invasion, proliferation, and shaping of the cancer microenvironment.^4,12,14,15,28^ These *in vitro* systems have been designed to establish IFF velocity magnitudes in line with those reported from a range of different platforms including computational modeling, extrapolation from *in vivo* data of bulk flow accumulation, and real-time or endpoint measurements of tracer molecules. Each of these methods has their strengths and limitations as discussed earlier, but overall, they lack the ability to examine the heterogeneity within the tumor and to directly measure interstitial fluid velocity using biotransport principles.

We utilized a commonly employed *in vitro* interstitial flow system to evaluate velocity patterns generated using our computational reconstruction approach and saw flow in the expected downward direction and on the same order of magnitude as the measured average velocity from the volumetric flow rate. These values changed as the matrix was synthesized to be more hydraulically resistant. Computed diffusivity measurements of the *in vitro* phantom as derived from the MRI method (supplementary material Fig. 3), though not the focus of the present study, were similarly on the same order of magnitude as previously reported values.^25,26^ Future work can validate this component of the model using more standard MRI methods such as diffusion-weighted MRI as a basis of comparison.^29^ Although the *in vitro* phantom serves well for assessing average values, spatial accuracy of the reconstructed velocity field could not be assessed in this setup. Therefore, we used *in silico* phantoms of three idealized flow scenarios to estimate the spatial accuracy of our reconstruction algorithm (Fig. 3). These experiments indicate overall good and spatially consistent performance for multidirectional flow fields of constant velocity magnitude, although based on our phantom results, we believe that we may be underestimating this magnitude by a factor of 2 [(Figs. 3(d), 3(e), 3(i), and 3(j)]. However, we caution about the interpretation of reconstructed velocity magnitude when the flow speed varies spatially [Figs. 3(m) and 3(n)]. In the latter scenario, we found the reconstructed velocity field to differ from the actual field not only by a spatially constant factor of magnitude but in a space-dependent manner, thus rendering comparisons between subregions difficult. As the fluid flow between the tumor and interstitium likely features a combination of the flow characteristics examined here, this phenomenon clearly limits the accuracy of our reconstruction algorithm when applied to *in vivo* data. Despite relatively large but very localized reconstruction errors in the velocity magnitude [Figs. 3(m) and 3(n)], our *in vitro* and *in silico* experiments indicate that reconstructed velocity fields are sufficiently accurate to estimate the bulk flow direction to approximately ±30°, even in boundary regions. This allows inward and outward flow to/from tumor from/to interstitium to be clearly distinguished in most situations.

The spatial accuracy at which the flow velocity fields can be reconstructed is intrinsically limited by the time interval between subsequent MR images, in our case approximately 3 minutes. Assuming an average IFF velocity magnitude of 2 *μ*m/s, we cannot expect to resolve characteristics of the flow field below a spatial resolution of approximately 400 *μ*m, corresponding to about 4 pixels. Therefore, spatial accuracy should not be affected by our choice to reconstruct the velocity in each pixel based on the intensity values from its 3 × 3 pixel neighborhood.

Since our reconstruction approach aims to estimate IFF flow indirectly from the change in concentration of an advected compound, its application is limited to situations in which such a change is detectable. In our experiments, parts of the tumor will either be saturated or void of contrast agent during a fraction of dynamic contrast-enhanced image acquisition. The lack of detectable concentration change in those regions results in less accurate reconstruction of the flow velocity field. This effect can be seen in the velocity field reconstructed from the MR phantom [Fig. 2(b)] where the velocity magnitude and direction are inconsistently estimated at the upper edge of the meniscus that remains void of contrast agent during most image acquisitions. Thus, the selection of appropriate concentrations and non-saturated locations is important to the overall accuracy of the reconstruction.

### Known contributors to transport in the brain tumor microenvironment

Interstitial flow has been tied to the increase in interstitial pressure within a confined tissue. It has been documented that larger tumors have higher interstitial fluid pressures,^11,30^ but we did not see a significant correlation between the tumor size and the average interstitial velocity magnitude. Although we would expect the fluid velocity to increase with the size of tumor, it is possible that in the confined space of the brain, there is an upper limit to the velocity magnitude, and thus, further work examining changes in intratumoral velocity during progression would yield these types of dynamic measurements. There are other factors that we did not examine that may contribute to changes in the average tumor velocity. The correlation between blood vessels and total fluid leakage and perfusion into tumors has been well-documented.^31–34^ The use of MRI to measure K_trans_ can be predictive of their tumor response to anti-angiogenic therapies, such as bevacizumab;^35^ however, these studies never examined the movement of this fluid once in the tumor. Leaky vasculature is required for entry of Gd contrast into the space, but our methodology specifically examines the movement of this agent once it has extravasated into the brain tumor, and thus while the initial signal is dependent on the vasculature density and permeability, only a change in signal over time is required for the overall measurement.

### The effects of the brain tumor microenvironment on interstitial fluid flow

As stated, high levels of intratumoral heterogeneity contribute to the inability to have definitive correlative findings between IFF and distinctive xenograft tumor models or size. Classically, interstitial fluid flow has been defined through modeling as moving in the outward direction from the tumor into the healthy tissue; however, we see that it is much more complex than that. Interstitial flow varies spatially throughout the tumor, and while this is unsurprising, the inward direction of flow in some border regions was unexpected. Indeed, these regions of inward flow correlated with lower Evans blue intensities [Fig. 6(e)], as did regions of lower average interstitial velocity magnitudes [Fig. 6(f)]. Evans blue has been used as a tracer of bulk drainage for decades both clinically and experimentally. We have used it in previous works to correlate regions of higher and lower flow based on assumptions that interstitial fluid drainage would correspond to the movement of this tracer and thus indicate the general trajectory of interstitial fluid velocities.^4,5^ Here, we demonstrate that these assumptions are valid by both depicting the magnitude of flow and to a lesser extent the direction of the flow corresponds to accumulation of this tracer. However, the relationship between this interstitial fluid drainage and bulk fluid drainage has not been specifically probed with our methodology; here, it stands to reason that this interstitial fluid eventually drains towards classical bulk flow structures within the brain.

Natural cerebrospinal fluid flow pathways in the brain may dictate tumor fluid behavior. In healthy tissue, bulk fluid flow pathways were identified almost 20 years ago along the white matter tracts,^36^ and natural movement of fluid towards these bulk pathways along white matter tracts may result in non-homogenous flow outward from the tumor. Similarly, in the glymphatic system, fluid drains along perivascular spaces,^37^ and thus tumor fluid flow, may be directed towards and along the nearby blood vessels leading to heterogeneous flow. Finally, tumor cells alter the surrounding extracellular matrix themselves or through the activation of surrounding stromal cells that then alter the matrix.^38^ These changes surrounding brain tumors include degradation of the native matrix^39^ and increases in non-native extracellular matrix (ECM) such as vitronectin,^40^ glycoproteins,^41^ Tenascin C,^42^ and Collagens.^43^ These irregular matrices can alter the permeability and hydraulic conductivity of the overall tissue leading to reduced or increased interstitial flow in regions.^44^ Thus, changes to the TME can alter flow and, in turn, the flow can alter the TME, similar to other mechanobiological forces.^45,46^ Understanding this feedback loop as it relates to the interstitial flow is important for dissecting out new mechanisms of cancer progression, not only in the brain but also in other organs as well.

### Implications in disease treatment and clinical outcomes

Magnetic resonance imaging is routinely used in GBM diagnosis, surgery, and postoperative monitoring and this straightforward imaging technique has been implemented in the clinical and pre-clinical settings for the study of GBM.^47^ Thereby, we have developed and used MRI methodology coupled with biotransport modeling to map interstitial flow in GBM xenografts. As IFF has been linked to increased invasion in GBM and areas of flow have correlated with invasive fronts in mouse models, we believe that our method could eventually act as a predictive indicator of regional invasion. This could allow physicians to aggressively target certain areas with surgery or anti-tumor chemotherapies, or benefit by identifying transport pathways to utilize during drug delivery. Finally, we can use MRI-derived IFF maps to determine if IFF is correlated with microenvironmental changes or activation of effectors that are leading to progression or worsened disease. Identification of such changes could act to identify new therapeutic targets. Assessment of IFF fields gives us the ability to study the tumor microenvironment in the context of an understudied biophysical force: interstitial flow, and thus, potentially open avenues for new understanding of glioblastoma.

## METHODS

### Cell culture and tumor inoculation

All animal experiments were approved by the University of Virginia Institutional Animal Care and Use committee under protocol number 4021. Briefly, Glioblastoma stem cells (GSCs) were cultured and maintained as previously described,^48^ and then steriotactically injected into 8–10 week old male Nonobese diabetic severe combined immunodeficiency (NOD SCID) mice with 15 000 GSCs [G2 (n = 3), G34 (n = 5), G62 (n = 4)] or 400 000 GSCs [G528 (n = 4)]. Injection coordinates were 2 mm lateral and posterior to bregma at a depth of 2.7 mm.

### MRI acquisition

Ten or eleven days post inoculation, mice were anesthetized, and tail vein catheters were inserted. Mice were then imaged with a 7T Clinscan system (Bruker, Ettlingen, Germany) equipped with a 30 mm diameter cylindrical RF coil. The presence of tumor was confirmed using a T2-weighted sequence with the following parameters: repetition time (TR) = 5500 ms, echo time (TE) = 650 ms, field of view (FOV) = 20 mm × 20 mm with a 192 × 192 matrix, slice thickness = 0.5 mm, number of slices = 30, and two averages per phase-encode step requiring a total acquisition time of about 5 min per sequence. Subsequent pre-contrast T1-weighted imaging was performed. A bolus injection of gadobenate dimeglumine was administered at a concentration of 0.3 mmol/kg in sterile saline (MultiHance, Bracco Diagnostics, Princeton, NJ). Following injection, a series of four post-contrast T1-weighted images were taken axially through the head with the following parameters: repetition time (TR) = 500 ms, echo time (TE) = 11 ms, field of view (FOV) = 20 mm × 20 mm with a 192 × 192 matrix, slice thickness = 0.7 mm, number of slices = 22, and two averages per phase-encode step requiring a total acquisition time of about 3 min per sequence. T1-weighted images were acquired for approximately 13 min post-injection.

### Computational model and equations

Gadolinium movement within the tumor and interstitium for multidimensional position ***x*** = (*x*, *y*, *z*) and time *t*, the model can be described by the following differential equation:

$$\frac{\partial \phi \left(x,t\right)}{\partial t}=\nabla \cdot \left[D\left(x,t\right)\nabla \phi \left(x,t\right)\right]-\nabla \cdot \left[\phi \left(x,t\right)u\left(x,t\right)\right],$$ (1)

where, at *spatial location* x and *time* t, we have ϕ(x,t) is the concentration of Gd $\left[\frac{\text{mmol}}{L^{3}}\right]$, D(x,t) is the isotropic diffusion coefficient $\left[\frac{L^{2}}{T}\right]$, and u(x,t) is the velocity field $\left[\frac{L}{T}\right]$.

Assuming that the intensity of the measured MRI signal, S=S(x, t), is directly proportional to Gd concentration, ϕ(x,t), the concentration in (1) can be replaced by MRI signal intensity. Due to the difference in spatial sampling within (104.17 *μ*m) and between (1.68 mm, slice thickness of 0.7 mm and 20% spacing between slices, 0.84 mm from the center of one to the center of the other) MR image slices, each axial slice (x-y plane) was considered independently. Therefore, the continuous-domain model of (1) was discretized in two spatial dimensions, using the forward-time, central-space (FTCS) finite difference method^49^ resulting in the following discrete-domain iterative model:

$$S^{\left(n+1\right)}(x,y)=S^{\left(n\right)}(x,y)+\frac{\Delta t}{\Delta s^{2}}D^{\left(n\right)}(x,y)\nabla^{2}S^{\left(n\right)}(x,y)-\frac{\Delta t}{2\Delta s}u^{\left(n\right)}(x,y)\cdot \nabla S^{\left(n\right)}(x,y),$$ (2)

where $S^{\left(n\right)}(x,y)$ is the measured MRI signal intensity in the axial (x-y) plane at discrete time n. Δt is the time interval between consecutive acquisitions of the same region of interest. Δs is the physical spacing between neighboring MRI pixels (in-plane resolution, identical in x and y directions). $\nabla^{2}$ is the discrete approximation of the Laplace operator obtained using the five-point kernel $\nabla^{2}\to \left[\begin{array}{ccc} 0 & 1 & 0\\ 1 & -4 & 1\\ 0 & 1 & 0 \end{array}\right].\;\nabla$ is the discrete approximation of the continuous gradient operator obtained using three-point kernels $\nabla_{x}\to \frac{1}{2}\left[\begin{array}{ccc} -1 & 0 & 1 \end{array}\right]$ and $\nabla_{y}\to \frac{1}{2}\left[\begin{array}{c} -1\\ 0\\ 1 \end{array}\right]$.

While (2) describes the expected temporal evolution of the intensity of each individual pixel given the parameters $D^{\left(n\right)}(x,y)$ and $u^{\left(n\right)}(x,y)$, we are interested in the inverse problem: We seek to identify the parameters $D^{\left(n\right)}(x,y)$ and $u^{\left(n\right)}(x,y)$, for each location (x,y) and each discrete time n from the temporal changes of the MRI signal under the assumption of the convective transport model in (1). As this system is underdetermined, we employ an optimization approach to identify the solution that minimizes the absolute difference between observed and model-predicted changes in MR signal intensity between two consecutive time points. Under the additional assumption that the diffusion coefficient and the velocity field are constant over the duration of the experiment [$D^{\left(n\right)}(x,y)$ *= D*(*x,y*) and $u^{\left(n\right)}(x,y)$ *= **u***(*x,y*)], (2) represents a system of *N-*1 equations (where N is the number of observation time points) and the optimization problem becomes

$$b_{m}=arg{min}_{b}\;\frac{1}{2}\;{\left\|A^{T}\cdot b-y\right\|}_{2}^{2}.$$ (3)

In (3), $y={\left[S^{\left(2\right)}-S^{\left(1\right)},S^{\left(3\right)}-S^{\left(2\right)},\ldots ,\;S^{\left(N\right)}-S^{\left(N-1\right)}\right]}^{T}$ is a vector of differences in MR signal intensity values between two consecutive temporal frames of a transversal slice, the matrix $A^{T}={\left[\frac{\Delta t}{\Delta s^{2}}\nabla^{2}S^{\left(n\right)},-\frac{\Delta t}{2\Delta s}S_{x}^{\left(n\right)},-\frac{\Delta t}{2\Delta s}S_{y}^{\left(n\right)}\right]}_{n=1,2,\ldots ,N-1}$ contains the image-derived values for each temporal frame, and $b={\left[D,\;u_{x},u_{y}\right]}^{T}$ represents a vector of the unknown parameters.

The solution of (3) is computed separately for each voxel in a selected MR slice after imposing the additional D≥0 constraint on the diffusion coefficient, yielding voxel-wise optimal values for each parameter of the transport model. Derived quantities (diffusion coefficient, velocity magnitude, velocity direction) are computed from these optimized parameter maps.

The optimization problem (3) was originally solved for each voxel independently of the behavior of its neighbors occasionally resulting in very sharp transition within the parameter maps. To provide a tighter coupling between the solutions evaluated at neighboring pixels, the optimization process was modified to extend y and A to include values from within a square region around the original voxel under analysis effectively utilizing all temporal values for all voxels within the region as part of the optimization process.

### Preparation of tissue culture insert phantoms

Collagen hydrogels composed of 1.8 mg/ml rat tail collagen I (Corning) and reduced growth factor basement membrane extract (BME, Cultrex) were seeded into 12 mm tissue culture inserts (Millipore, Burlington, MA). A flow condition is created by adding media atop the gel creating a pressure head. [Fig. 2(b)].^24^ Volumetric flow rates and average superficial velocity were experimentally determined over a period of 30 min as defined by the following equation:

$$v_{avg}=\frac{Q}{A},$$ (4)

where Q is the volumetric flow rate as determined by manually collecting and measuring the total volume flowed through the gel within a given time. A is the cross-sectional area of the tissue culture insert.

### *In silico* phantoms

*In silico* models were created to investigate the accuracy of the proposed reconstruction algorithm and compare estimated to actual model parameters by computationally solving (FENICS) the forward model (1) for specific flow scenarios. We examined three scenarios: standard scenarios: bidirectional flow [Fig. 3(b)] multidirectional (360°) flow of spatially constant magnitude [Fig. 3(g)], which replicates an idealized tumor model with pressure in the outward direction, and unidirectional flow of spatially varying magnitude [Fig. 3(l)].

For each scenario, an *in silico* phantom was created by defining an initial distribution of solute (Gd) *ϕ*(x, y, t = 0) [Figs. 3(a), 3(f), and 3(k)] and computationally simulating its time-evolution according to (1) for prescribed velocity fields, ***u***(*x*, *y*), and spatially constant diffusivity, *D*(*x*, *y*) = *D*. From the solution of this forward model, maps of Gd concentration *ϕ*(x, y, t = t_n_) were extracted at multiple evaluation time points t_n_, analogous to MRI scenarios with spatiotemporally evolving contrast enhancement. Flow velocity was estimated from these synthetic images by applying our reconstruction algorithm. Finally, we compared the direction and magnitude of prescribed and reconstructed velocity fields for each simulated flow scenario.

The forward model describing the time evolution of the concentration was solved in 2D using the Finite-Element Method. All simulations assumed a spatially constant isotropic diffusion coefficient (1 × 10^−4^ cm^2^/s)^26^ and were solved in time steps dt = 0.01 s. Solutions were extrapolated onto a 50 × 50 pixel regular grid to mimic the image size of large tumors at typical small-animal MR in-plane resolution.

### Tissue post-processing

Following MRI acquisition (ten or eleven days after inoculation), animals were injected with Evans blue dye administered intravenously. The following day, animals were euthanized by intracardial perfusion with saline and brains were cryoembedded and sectioned at 12 *μ*m. Sections at varying depths within the tumor were immunostained for mouse anti-human nuclei (clone 235-1, Millipore) and 4',6-Diamidino-2-Phenylindole, Dihydrochloride (DAPI, Sigma). Whole section scans were taken using an EVOS FL Auto 2.0 and images processed using Photoshop for figures. Raw images were imported into ImageJ, and four to five user-defined regions of Evans blue positivity were selected and intensity was measured using integrated density. Regions were approximately 0.49 mm^2^, and the integrated density in each region was normalized to the tumor maximum. Regions were defined as ‘high flow’ if the integrated density was greater than 2/3 the maximum for the tumor. Using DAPI and anti-human nuclei staining to identify tumor size and location, sections were matched to closest corresponding MRI slice. The velocity was computed in the matched regions, and average velocity and direction were determined. Briefly, flow was considered ‘outward’ if the average direction was ±45 degrees from the normal to the tumor border, and ‘non-outward’ if the direction was parallel. Statistical analysis was performed in GraphPad to compare Evans blue intensity values with velocity direction and magnitude. Wilcoxon matched-pairs signed-rank tests were performed, to correct for non-parametric distributions, to compare outward vs non-outward flow, and low and high flow for each tumor.

## SUPPLEMENTARY MATERIAL

See supplementary material for the summary data and histograms of the *in vitro* and *in vivo* calculated diffusivities, as well as the histograms of the *in vivo* calculated velocity magnitude for the individual tumor models.

## References

[1] P. A. Kenny and M. J. Bissell , Int. J. Cancer 107, 688 (2003). 10.1002/ijc.11491 14566816 PMC2933180

[2] M. A. Swartz , N. Iida , E. W. Roberts , S. Sangaletti , M. H. Wong , F. E. Yull , L. M. Coussens , and Y. A. DeClerck , Cancer Res. 72, 2473 (2012). 10.1158/0008-5472.CAN-12-0122 22414581 PMC3653596

[3] S. Xia , B. Lal , B. Tung , S. Wang , C. R. Goodwin , and J. Laterra , Neuro. Oncol. 18, 507 (2015). 10.1093/neuonc/nov171 26320116 PMC4799677

[4] J. M. Munson , R. V. Bellamkonda , and M. A. Swartz , Cancer Res. 73, 1536 (2013). 10.1158/0008-5472.CAN-12-2838 23271726

[5] K. M. Kingsmore , D. K. Logsdon , D. H. Floyd , S. M. Peirce , B. W. Purow , and J. M. Munson , Integr. Biol. 8, 1246 (2016). 10.1039/C6IB00167J 27775742

[6] A. D. Shah , M. J. Bouchard , and A. C. Shieh , PLoS One 10, e0142337 (2015). 10.1371/journal.pone.0142337 26560447 PMC4641731

[7] U. Haessler , J. C. Teo , D. Foretay , P. Renaud , and M. A. Swartz , Integr. Biol. 4, 401 (2012). 10.1039/C1IB00128K 22143066

[8] M. E. Fleury , K. C. Boardman , and M. A. Swartz , Biophys. J. 91, 113 (2006). 10.1529/biophysj.105.080192 16603487 PMC1479084

[9] J. D. Shields , M. E. Fleury , C. Yong , A. A. Tomei , G. J. Randolph , and M. A. Swartz , Cancer Cell 11, 526 (2007). 10.1016/j.ccr.2007.04.020 17560334

[10] Y. L. Huang , C. Tung , A. Zheng , B. J. Kim , and M. Wu , Integr. Biol. 7, 1402 (2015). 10.1039/C5IB00115C PMC4630101 26235230

[11] Y. Boucher , H. Salehil , B. Witwerl , G. Harsh , and R. Jain , Br. J. Cancer 75, 829 (1997). 10.1038/bjc.1997.148 9062403 PMC2063404

[12] H. Qazi , Z. D. Shi , and J. M. Tarbell , PLoS One 6, e20348 (2011). 10.1371/journal.pone.0020348 21637818 PMC3102715

[13] T. Hompland , C. Ellingsen , K. M. Øvrebø , and E. K. Rofstad , Cancer Res. 72, 4899 (2012). 10.1158/0008-5472.CAN-12-0903 23027087

[14] W. J. Polacheck , J. L. Charest , and R. D. Kamm , Proc. Natl. Acad. Sci. 108, 11115 (2011). 10.1073/pnas.1103581108 21690404 PMC3131352

[15] A. C. Shieh , H. A. Rozansky , B. Hinz , and M. A. Swartz , Cancer Res. 71, 790 (2011). 10.1158/0008-5472.CAN-10-1513 21245098

[16] S. R. Chary and R. K. Jain , Proc. Natl. Acad. Sci. U. S. A. 86, 5385 (1989). 10.1073/pnas.86.14.5385 2748592 PMC297627

[17] T. P. Butler , F. H. Grantham , and P. M. Gullino , Cancer Res. 35, 3084 (1975). 1182701

[18] H. Dafni , T. Israely , Z. M. Bhujwalla , L. E. Benjamin , and M. Neeman , Cancer Res. 62, 6731 (2002). 12438274

[19] A. P. Pathak , D. Artemov , B. D. Ward , D. G. Jackson , M. Neeman , and Z. M. Bhujwalla , Cancer Res. 65, 1425 (2005). 10.1158/0008-5472.CAN-04-3682 15735030

[20] L. J. Liu , S. L. Brown , J. R. Ewing , B. D. Ala , K. M. Schneider , and M. Schlesinger , PLoS One 11, e0140892 (2016). 10.1371/journal.pone.0140892 27467886 PMC4965107

[21] M. Zhao , L.-L. Guo , N. Huang , Q. Wu , L. Zhou , H. Zhao , J. Zhang , and K. Fu , Oncol. Lett. 14, 5418 (2017). 10.3892/ol.2017.6895 29113174 PMC5656018

[22] J. Bolcaen , B. Descamps , M. Acou , K. Deblaere , C. Van den Broecke , T. Boterberg , C. Vanhove , and I. Goethals , Mol. Imaging Biol. 19, 857 (2017). 10.1007/s11307-017-1071-0 28303489

[23] A. N. V. Dehkordi , A. Kamali-Asl , N. Wen , T. Mikkelsen , I. J. Chetty , and H. Bagher-Ebadian , NMR Biomed. 30, e3739 (2017). 10.1002/nbm.3739 28543885

[24] A. R. Harris , J. X. Yuan , and J. M. Munson , Methods 134–135, 20 (2017). 10.1016/j.ymeth.2017.12.010 PMC5815918 29258924

[25] D. K. Logsdon , G. F. Beeghly , and J. M. Munson , Cell. Mol. Bioeng. 10, 463 (2017). 10.1007/s12195-017-0498-3 PMC6816789 31719872

[26] S. Ramanujan , A. Pluen , T. D. McKee , E. B. Brown , Y. Boucher , and R. K. Jain , Biophys. J. 83, 1650 (2002). 10.1016/S0006-3495(02)73933-7 12202388 PMC1302261

[27] K.-C. Wei , P.-C. Chu , H.-Y. J. Wang , C.-Y. Huang , P.-Y. Chen , H.-C. Tsai , Y.-J. Lu , P.-Y. Lee , I.-C. Tseng , L.-Y. Feng , P.-W. Hsu , T.-C. Yen , and H.-L. Liu , PLoS One 8, e58995 (2013). 10.1371/journal.pone.0058995 23527068 PMC3602591

[28] A. C. Shieh , Ann. Biomed. Eng. 39, 1379 (2011). 10.1007/s10439-011-0252-2 21253819

[29] S. Lope-Piedrafita , M. L. Garcia-Martin , J.-P. Galons , R. J. Gillies , and T. P. Trouard , NMR Biomed. 21, 799 (2008). 10.1002/nbm.1256 18470959 PMC2857329

[30] Y. Boucher , L. T. Baxter , and R. K. Jain , Cancer Res. 50, 4478 (1990). 2369726

[31] H. J. Aronen , I. E. Gazit , D. N. Louis , B. R. Buchbinder , F. S. Pardo , R. M. Weisskoff , G. R. Harsh , G. R. Cosgrove , E. F. Halpern , and F. H. Hochberg , Radiology 191, 41 (1994). 10.1148/radiology.191.1.8134596 8134596

[32] O. Tynninen , H. J. Aronen , M. Ruhala , A. Paetau , K. Von Boguslawski , O. Salonen , J. Jääskeläinen , and T. Paavonen , Invest. Radiol. 34, 427 (1999). 10.1097/00004424-199906000-00007 10353036

[33] E. A. Knopp , S. Cha , G. Johnson , A. Mazumdar , J. G. Golfinos , D. Zagzag , D. C. Miller , P. J. Kelly , and I. I. Kricheff , Radiology 211, 791 (1999). 10.1148/radiology.211.3.r99jn46791 10352608

[34] S. Cha , G. Johnson , Y. Z. Wadghiri , O. Jin , J. Babb , D. Zagzag , and D. H. Turnbull , Magn. Reson. Med. 49, 848 (2003). 10.1002/mrm.10446 12704767

[35] R. T. Ullrich , J. F. Jikeli , M. Diedenhofen , P. Böhm-Sturm , M. Unruh , S. Vollmar , and M. Hoehn , PLoS One 6, e19592 (2011). 10.1371/journal.pone.0019592 21573168 PMC3088680

[36] C. P. Geer and S. A. Grossman , J. Neurooncol. 32, 193 (1997). 10.1023/A:1005761031077 9049880

[37] J. J. Iliff , M. Wang , Y. Liao , B. A. Plogg , W. Peng , G. A. Gundersen , H. Benveniste , G. E. Vates , R. Deane , S. A. Goldman , E. A. Nagelhus , and M. Nedergaard , Sci. Transl. Med. 4, 147ra111 (2012). 10.1126/scitranslmed.3003748 PMC3551275 22896675

[38] P. Lu , V. M. Weaver , and Z. Werb , J. Cell Biol. 196, 395 (2012). 10.1083/jcb.201102147 22351925 PMC3283993

[39] C.-F. Yu , F.-H. Chen , M.-H. Lu , J.-H. Hong , and C.-S. Chiang , Br. J. Cancer 117, 1828 (2017). 10.1038/bjc.2017.362 29065106 PMC5729475

[40] C. L. Gladson and D. A. Cheresh , J. Clin. Invest. 88, 1924 (1991). 10.1172/JCI115516 1721625 PMC295768

[41] J. Zámecník , L. Vargová , A. Homola , R. Kodet , and E. Syková , Neuropathol. Appl. Neurobiol. 30, 338 (2004). 10.1046/j.0305-1846.2003.00541.x 15305979

[42] Y. A. Miroshnikova , J. K. Mouw , J. M. Barnes , M. W. Pickup , J. N. Lakins , Y. Kim , K. Lobo , A. I. Persson , G. F. Reis , T. R. McKnight , E. C. Holland , J. J. Phillips , and V. M. Weaver , Nat. Cell Biol. 18, 1336 (2016). 10.1038/ncb3429 27820599 PMC5361403

[43] L. S. Payne and P. H. Huang , Mol. Cancer Res. 11, 1129 (2013). 10.1158/1541-7786.MCR-13-0236 23861322 PMC3836242

[44] P. A. Netti , D. A. Berk , M. A. Swartz , A. J. Grodzinsky , and R. K. Jain , Cancer Res. 60, 2497 (2000). 10811131

[45] J. L. Leight , A. P. Drain , and V. M. Weaver , Annu. Rev. Cancer Biol. 1, 313 (2017). 10.1146/annurev-cancerbio-050216-034431

[46] P. Cirri and P. Chiarugi , Cancer Metastasis Rev. 31, 195 (2012). 10.1007/s10555-011-9340-x 22101652

[47] P. L. Kubben , K. J. ter Meulen , O. E. Schijns , M. P. ter Laak-Poort , J. J. van Overbeeke , and H. van Santbrink , Lancet Oncol. 12, 1062 (2011). 10.1016/S1470-2045(11)70130-9 21868286

[48] J. Lee , S. Kotliarova , Y. Kotliarov , A. Li , Q. Su , N. M. Donin , S. Pastorino , B. W. Purow , N. Christopher , W. Zhang , J. K. Park , and H. A. Fine , Cancer Cell 9, 391 (2006). 10.1016/j.ccr.2006.03.030 16697959

[49] J. H. Ferziger and M. Perić , *Computational Methods for Fluid Dynamics* ( Springer, Berlin/Heidelberg, 2002).

